# Assessing the Potential of Gallic Acid and Methyl Gallate to Enhance the Efficacy of β-Lactam Antibiotics against Methicillin-Resistant *Staphylococcus aureus* by Targeting β-Lactamase: In Silico and In Vitro Studies

**DOI:** 10.3390/antibiotics12111622

**Published:** 2023-11-13

**Authors:** Pimsumon Jiamboonsri, Chatchakorn Eurtivong, Sompit Wanwong

**Affiliations:** 1 Faculty of Medicine, King Mongkut’s Institute of Technology Ladkrabang, 1 Chalongkrung Road, Ladkrabang, Bangkok 10520, Thailand; 2 Department of Pharmaceutical Chemistry, Faculty of Pharmacy, Mahidol University, 447 Si Ayutthaya Road, Ratchathewi, Bangkok 10400, Thailand; chatchakorn.eur@mahidol.edu; 3 Materials Technology Program, School of Energy, Environment and Materials, King Mongkut’s University of Technology Thonburi, 126 Pracha Uthit Road, Thung Khru, Bangkok 10140, Thailand; sompit.wan@kmutt.ac.th

**Keywords:** gallic acid, methyl gallate, methicillin-resistant *Staphylococcus aureus*, β-lactamase, β-lactam antibiotics

## Abstract

Methicillin-resistant *Staphylococcus aureus* (MRSA), a global health concern, has prompted research into antibiotic adjuvants as a potential solution. Although our group previously reported the enhancing effects of gallic acid (GA) and methyl gallate (MG) on penicillin G activity against MRSA, the synergistic potential with other β-lactam antibiotics and the underlying mechanism have not been fully explored. Therefore, this study primarily aimed to investigate the antibacterial synergism with β-lactam antibiotics through disc diffusion, checkerboard, and time–kill assays. The β-lactamase inhibition was also examined through both molecular modeling and in vitro experiments. Additionally, bacterial morphology changes were studied using a scanning electron microscopy (SEM). The results revealed that both GA and MG exhibited anti-MRSA activity and showed indifferent effects when combined with β-lactam antibiotics against methicillin susceptible *S. aureus* (MSSA). Interestingly, MG demonstrated synergism with only the β-lactamase-unstable antibiotics against MRSA with the lowest fractional inhibitory concentration (FIC) indexes of ≤3.75. However, GA and MG exhibited weak β-lactamase inhibition. Furthermore, GA, MG, and the combination with ampicillin induced the morphological changes in MRSA, suggesting a possible mechanism affecting the cell membrane. These findings suggest that MG could potentially serve as an adjunct to β-lactam antibiotics to combat MRSA infections.

## 1. Introduction

Penicillin, discovered in the 1920s, has become one of the most well-known antibiotics for bacterial eradication by inhibiting cell wall biosynthesis [1,2]. It is categorized as a member of the β-lactam antibiotic class because of its β-lactam-based structure [3]. Generally, β-lactam antibiotics are the first-line agents prescribed for treating common bacterial infections and for managing critically ill patients with septic shock due to their safety profile [4]. They are typically used to target a broad spectrum of both Gram-positive and Gram-negative bacteria. Among Gram-positive bacteria, *S. aureus* is a prominent human skin pathogen. It can cause a range of illnesses, from common skin infection to life-threatening bacterial diseases, such as pneumonia, meningitis, osteomyelitis, endocarditis, and toxic shock syndrome [5]. Although the introduction of β-lactam antibiotics significantly improved the prognosis of patients with severe staphylococcal infections, *S. aureus* developed resistance to penicillin in 1942 through the production of penicillinase [6]. To counter this resistance mechanism, methicillin, oxacillin and cloxacillin, which are isoxazolyl penicillins, were developed to enhance stability against staphylococcal β-lactamase. Additionally, the aminopenicillins, including ampicillin, and amoxicillin, were introduced as the first semi-synthetic penicillins against Gram-negative bacteria following the launch of penicillinase-stable antibiotics. However, despite their initial success against staphylococcal infections, MRSA emerged in 1979–1980, subsequently becoming a global public health problem and a significant contributor to both hospital-acquired and community infections [1,7].

Under these circumstances, combination therapy has been extensively investigated to control MRSA, alongside the pursuit of novel antibiotic agents, which typically requires significant time for its development and approval by the FDA [8]. The antibiotic adjuvant can encompass either different class of antibiotics, or other agents lacking antimicrobial activity but enhancing the efficacy of antibiotic drugs [9]. For example, β-lactamase inhibitors, such as clavulanic acid, sulbactam, and tazobactam, are co-administered agents designed to prevent β-lactam breakdown by serine β-lactamases, thereby restoring the effectiveness of β-lactam antibiotics [10].

GA and MG, which are secondary metabolites of plants generally found in many foods and beverages, have gained attention due to their favorable safety information [11,12]. GA and MG have been reported to offer many health benefits, including antioxidant [13], anti-cancers [14,15], anti-inflammatory [16,17] and antimicrobial effects [18,19,20]. Their pharmacological activities have been documented to be associated with enzyme inhibition because of the protein-binding capabilities of phenolic compounds [21]. For example, Sharanya et al. (2018) showed that MG could inhibit lipoxygenase activity through wet-laboratory experiments and in silico studies, highlighting its potential anti-inflammatory therapy [16]. Similarly, GA shows promise in inflammatory therapy due to its inhibitory effect on phospholipase A2 [17]. However, the inhibition of bacterial enzymes or virulence by other phenolic compounds have been reported to be either associated or not associated with their antibacterial activity [22]. Bi et al. (2016) reported that chlorogenic acid, a natural phenolic compound, served as a potent inhibitor against sortases, which are Gram-positive bacterial transpeptidases responsible for covalently attaching secreted proteins to the peptidoglycan cell wall [23]. It exhibited the inhibitory concertation (IC_50_) of 34 μg/mL, but it showed weak suppression of *S. aureus* growth with the minimum inhibitory concentration (MIC) greater than 1024 μg/mL [23]. On the other hand, epicatechin gallate, possessing antibacterial activity with an MIC of 100 μg/mL, could inhibit penicillinase with an IC_50_ of 10 μg/mL, leading to the restoration of penicillin’s antibacterial activity [24].

In our previous study, we demonstrated the antibacterial activities of GA and MG, as well as their ability to enhance penicillin G activity against clinical MRSA isolates [25]. However, there are limited available data regarding their synergistic effects, and the role of β-lactamase in enhancing antibiotic activity remains unclear. Therefore, this study mainly aimed to investigate antibacterial synergy between GA, MG, and other β-lactam antibiotics against both MSSA and MRSA, using disc diffusion, checkerboard, and time–killing assays. The possible mechanism of β-lactamase inhibition was explored through in silico molecular modelling and in vitro enzyme inhibition assays. Additionally, the effects of GA and MG on bacterial membrane morphology were also observed by SEM.

## 2. Results

### 2.1. Antibacterial Susceptibility

Table 1 demonstrates the MIC and the minimum bactericidal concentration (MBC) of phenolic compounds and the β-lactam antibiotic against *S. aureus* ATCC 25923 and 43300, known as MSSA and MRSA, respectively. In the case of the phenolic compounds, GA exhibited higher potency against MSSA than MRSA, with approximately a 2-fold difference (200 and 400 μg/mL, respectively, for MIC, and 400 and >400 μg/mL, respectively, for MBC). Furthermore, the potency of GA against MSSA and MRSA was higher than that of MG by at least 4-fold. Remarkably, MG showed equivalent MIC and MBC values for both bacterial strains at 1600 and 3200 μg/mL, respectively.

Among the β-lactam antibiotics used to combat MSSA, the order of potency for MICs was as follows: penicillin G (0.0625 μg/mL) > ampicillin (0.125 μg/mL) > amoxicillin and cloxacillin (0.25 μg/mL), and this order was consistent for MBCs as well. Additionally, the MBC values were two-fold higher than the corresponding MIC values. In contrast, all β-lactam antibiotics exhibited higher MICs and MBCs against MRSA compared to MSSA, with at least a two-fold difference. The order of potency for MICs against MRSA was as follows: cloxacillin (0.5 μg/mL) > ampicillin, amoxicillin, and penicillin G (62.5 μg/mL). The order of potency considered by MBC values were as follows: cloxacillin (1 μg/mL) > penicillin G (125 μg/mL) > ampicillin and amoxicillin (250 μg/mL). No antibacterial activity of the used solvent could be observed.

### 2.2. Enhancing Effects of GA and MG on Anti-MRSA Activity of β-Lactam Antibiotics

#### 2.2.1. Disc Diffusion Assay

**Figure 1:**
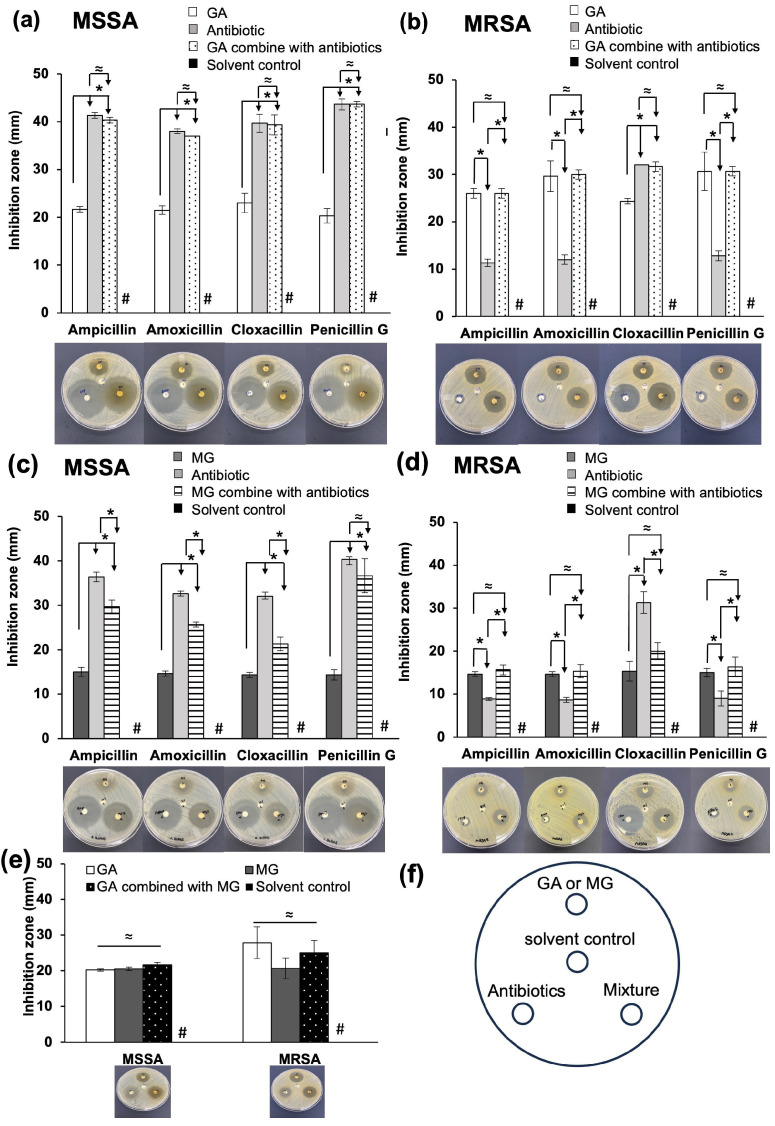
The inhibition zones of either GA or MG and its combination with ampicillin, amoxicillin, cloxacillin and penicillin G against (**a**,**c**) MSSA (*S. aureus* ATCC 25923), and (**b**,**d**) MRSA (*S. aureus* ATCC 43300). (**e**) The inhibition zones of the MG combined with GA against MSSA and MRSA. (**f**) Schematic diagram showing position of GA or MG, antibiotics, solvent control, and mixture of compounds on the agar plate. Data at each point indicate the mean ± standard deviation (S.D.) of the triplicate samples. * Significant difference, *p* < 0.05; ≈ no significant difference, *p* > 0.05; # indicates no inhibition zone.

The enhancing effects of GA and MG on the antibacterial activities of β-lactam antibiotics were primarily assessed by disc diffusion assay at fixed concentrations. As displayed in Figure 1a–d, GA and MG demonstrated similar inhibition patterns against MSSA and MRSA. At a concentration of 10 mg/disc, both phenolic compounds demonstrated significantly smaller mean inhibition zones against MSSA compared to all tested β-lactams at 10 μg/disc (*p <* 0.05). However, their inhibition zones against MSSA were comparable to those against MRSA.

In the case of the β-lactam antibiotics illustrated in Figure 1a–d, the inhibition zones of the β-lactamase-unstable antibiotic, including ampicillin, amoxicillin, and penicillin G, against MSSA were in a range of 32.67 ± 0.58 to 43.67 ± 1.15 mm and noticeably reduced by more than 2-fold compared to those against MRSA (8.67 ± 0.58 to 12.83 ± 1.04 mm). In contrast, the inhibition zone of cloxacillin, a β-lactamase-stable antibiotic, showed only a slight decrease when tested against MRSA (32.00 ± 1.00 to 39.67 ± 1.89 mm) in comparison to its performance against MSSA (31.33 ± 2.52 to 32.00 ± 0.00 mm).

When MSSA was treated with the combination of GA and the β-lactam antibiotics (Figure 1a), the average diameters of their clear zones were not significantly different from those of the individual β-lactams (*p* > 0.05), indicating an indifferent effect. However, the combination of GA and the β-lactam antibiotic yielded different results against MRSA (Figure 1b). When GA was combined with the β-lactamase-unstable antibiotics, the clear zone diameters were not different from those of a single GA (*p* > 0.05) but were significantly higher than those of individual antibiotics (*p* < 0.05). Conversely, when GA was combined with cloxacillin, there was no significant difference between the inhibition zone of the combination and that of cloxacillin alone, suggesting the possible involvement of β-lactamase in the mechanism of GA.

In agreement with the results obtained with GA, the combination of MG and penicillin G against MSSA showed an indifferent inhibition zone to that of penicillin G alone (Figure 1c). However, when MG was combined with ampicillin, amoxicillin and cloxacillin against MSSA, it significantly reduced inhibition zones of the combinations compared to the individual β-lactam antibiotics (*p* < 0.05), indicating interference in antibiotic activity. Regarding MRSA (Figure 1d), MG exhibited a similar antibacterial trend to GA. The clear zone diameters of combinations between MG and the β-lactamase-unstable antibiotics were not significant from those of MG alone (*p* > 0.05), but they were significantly higher than those of antibiotics alone (*p* < 0.05). Additionally, when MG was combined with cloxacillin, its inhibition zone was smaller than that of individual cloxacillin, but not different from that of MG alone.

As displayed in Figure 1e, there are no significant differences (*p* > 0.05) among the diameters of individual GA, MG, and their combination against both bacterial strains, indicating the absence of any significant antibacterial interaction between GA and MG.

#### 2.2.2. Synergistic Effects of GA and MG on the Anti-MRSA Activity of β-Lactam Antibiotics

The assessment of antibacterial synergism between either GA or MG and the β-lactam antibiotics were determined by checkerboard method. Table 2 summarizes the lowest FIC indexes for each combination against MSSA and MRSA. For the combination of GA and MG, the FIC indexes indicated an additive activity against both strains with a similar value of 0.63. Moreover, both GA and MG displayed indifferent activity to all tested β-lactams against MSSA, as evidenced by an FIC index of 1.03.

When the phenolic compounds were combined with ampicillin, amoxicillin, and penicillin G against MRSA, both GA, and MG were able to decrease the MIC of these antibiotics by at least 2-fold. Moreover, the FIC indexes of these antibiotics in combination with GA and MG indicated the additive and synergistic effect, respectively. Among these combinations, the lowest FIC index of 0.313 was observed for MG combined with ampicillin, followed by the combinations with amoxicillin and penicillin G (FIC index of 0.375). Interestingly, neither GA nor MG could enhance the activity of cloxacillin. The FIC indexes of both GA and MG showed indifferent activity when combining with cloxacillin.

#### 2.2.3. Time–Kill Curves

**Figure 2:**
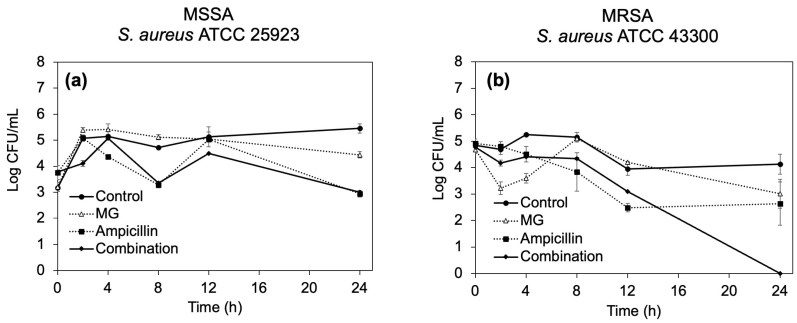
Time–kill curves of (**a**) *S. aureus* ATCC 25923 and (**b**) ATCC 43300 after treatment with MG, ampicillin, and their combination at MIC. Each symbol indicates the mean ± S.D. of triplicate samples.

Based on the lowest FIC indexes, the combination of MG and ampicillin was selected for further investigation to observe a dynamic synergistic effect against *S. aureus* ATCC 25923 and 43300, as displayed in Figure 2. After treatment MSSA for 24 h (Figure 2a), MG at its MIC suppressed the bacterial growth by approximately 1-log_10_-fold reduction compared with the control, whereas treatment with ampicillin alone and in combination with MG showed a similar reduction in bacterial number, approximately a 2.5-log_10_-fold decrease compared with the control, indicating an indifferent effect between MG and ampicillin.

On the other hand, when MG or ampicillin alone at their respective MIC level was used against MRSA, bacterial growth was reduced by approximately 1.13-log_10_-fold compared with the control after 24 h (Figure 2b). However, no surviving bacteria were observed after treatment with the combination. The reduction in bacterial numbers after treatment with the combination was calculated as 2.64-log_10_-fold reduction compared with ampicillin alone, indicating the synergism of MG and ampicillin against MRSA.

### 2.3. β-Lactamase Inhibition

#### 2.3.1. In Silico Molecular Modelling

**Figure 3:**
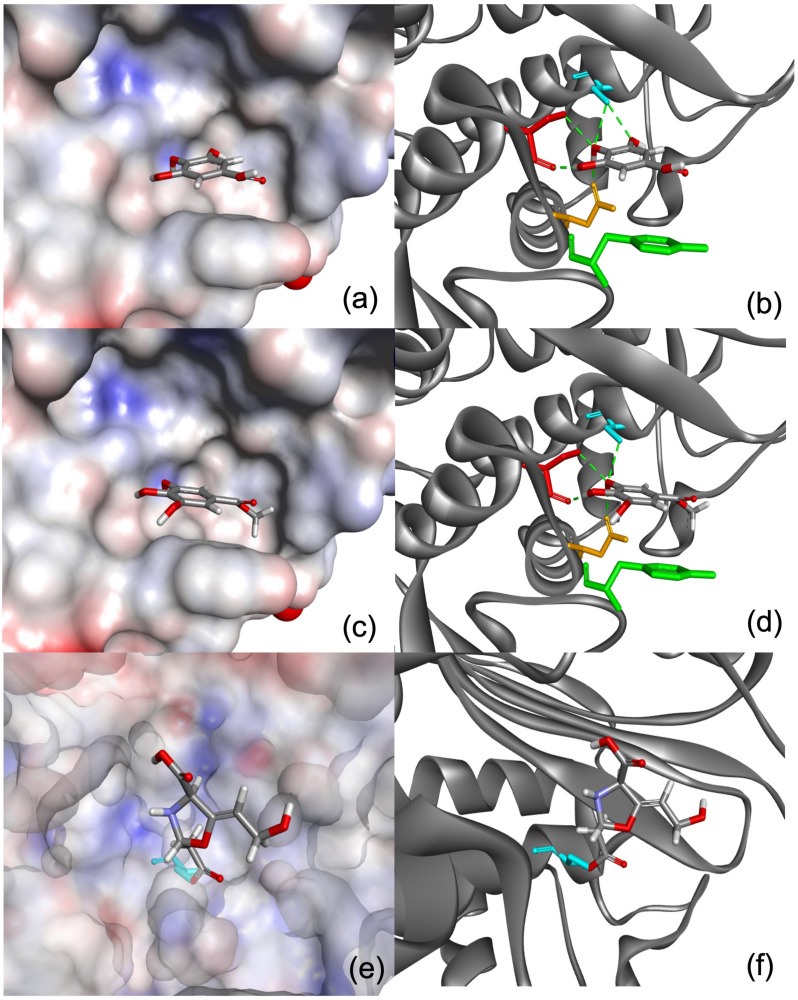
(**a**,**b**) The predicted binding modes of GA, (**c**,**d**) MG and (**e**,**f**) clavulanic acid. The protein surfaces in a, c and e are rendered in which partial positive, negative, and neutral charges are depicted as red, blue, and grey areas. In b, d and f, the protein structures are represented as grey ribbons. Amino acid residues Tyr105 (green), Ser70 (turquoise), Ser130 (red) and Asn132 (orange) are shown as sticks representation, and hydrogen bond formations are depicted as green dotted lines.

The chemical structures of GA and MG were docked to the enzyme active site. GA and MG scored comparably, at 32.64 and 36.89, respectively, whereas the covalent inhibitor, clavulanic acid, scored 101.12. The scores are relatively in line with the activities shown in the assay results. The predicted binding modes of the GA and clavulanic acid are shown in Figure 3. It is predicted that GA is able to form hydrogen bond interactions with residues Ser70, Ser130 and Asn132, and hydrophobic interactions are expected to primarily form with a nearby Tyr105. However, given that the spacious binding site is mainly hydrophobic, it is unlikely that small polar substrates such as GA and its ester derivative, MG, will bind strongly to the enzyme, which explains the low docking scores, and relatively mild and inactivity of the two substrates. In contrast, clavulanic acid strongly inhibits the enzyme-binding site via formation of a covalent link with Ser70, which is evidently apparent from its high docking score, indicating its strong potency.

#### 2.3.2. In Vitro β-Lactamase Inhibition

**Figure 4:**
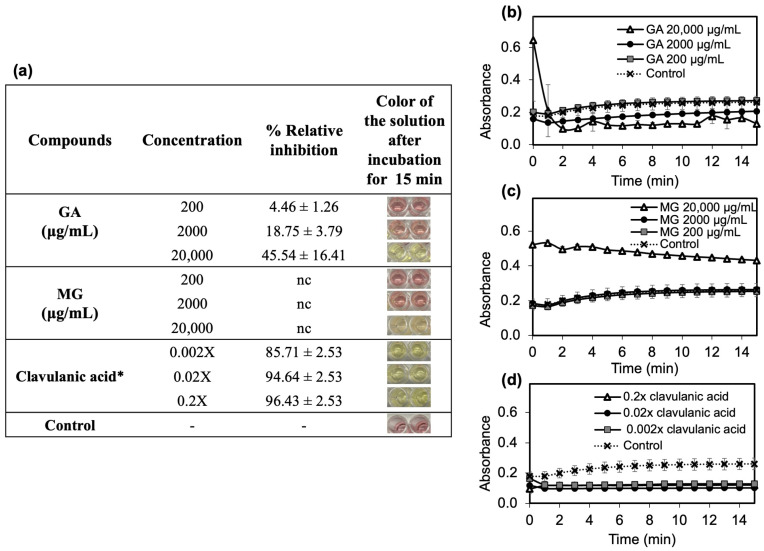
(**a**) The relative inhibitory effects of GA, MG, and the reference clavulanic acid on β-lactamase. The kinetic of the nitrocefin-product formation plotting at wavelength (490 nm) vs. incubation time after β-lactamase incubated with (**b**) GA, (**c**) MG, and (**d**) clavulanic acid. * The concentration of clavulanic acid demonstrates as 0.002, 0.02 and 0.2-folds from the original concentration. Each symbol indicates mean ± S.D. (*n* = 2), nc = the % relative inhibition could not be calculated.

The ability of GA and MG to inhibit β-lactamase was evaluated using an enzymatic screening assay kit. The hydrolysis of a yellow nitrocefin substrate generates a red product at an OD 490 nm, which is directly proportional to the amount of enzyme activity. As presented in the inserted table in Figure 4a, the addition of GA or MG at 200 and 2000 μg/mL resulted in the red solution, whereas the presence of GA or MG at 20,000 μg/mL provided a yellow solution. This outcome suggests that GA and MG could inhibit the β-lactamase activity at high concentration of 20,000 μg/mL. However, the inhibition capacity of GA and MG showed different patterns. The addition of GA caused the lower formation rate of nitrocefin product than the control in a concentration-dependent manner (Figure 4b). The relative inhibition ranged from 4.46 to 45.54% with an increasing concentration of GA from 200 to 20,000 μg/mL. On the other hand, the formation rates of nitrocefin product in the present of MG at 200 and 2000 μg/mL were not significantly different from the control, indicating no enzymatic inhibition (Figure 4c). Therefore, GA and MG at 20,000 μg/mL were considered weak β-lactamase inhibitors at both active and allosteric sites, especially when compared to clavulanic acid, which demonstrated the relative inhibitory effect of more than 85.7%

### 2.4. SEM

**Figure 5:**
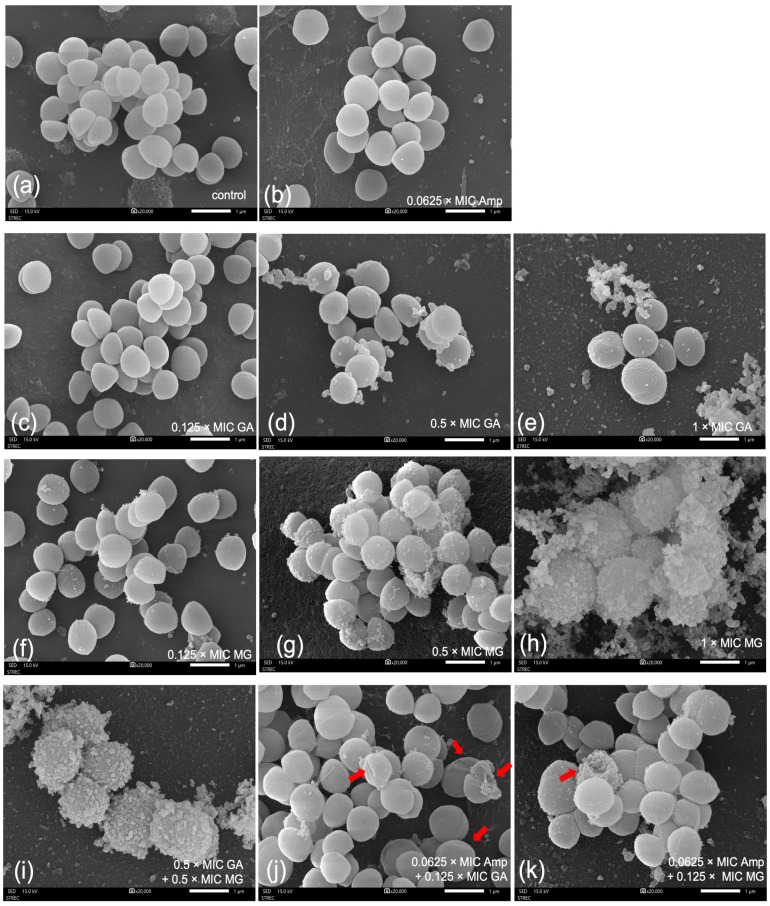
Scanning electron micrographs (20,000×) of *S. aureus* ATCC 43300 at 12 h after treatment with (**a**) control, (**b**) ampicillin (Amp) at 0.0625 × MIC, (**c**–**e**) GA at 0.125, 0.5 and 1 × MICs, (**f**–**h**) MG at 0.125, 0.5 and 1 × MICs, (**i**) the combination of GA and MG at 0.5 × MICs, (**j**) the combination of ampicillin at 0.0625 × MIC and GA at 0.125 × MIC, and (**k**) the combination of ampicillin at 0.0625 × MIC and MG at 0.125 × MIC.

Figure 5 shows representative SEM images of MRSA ATCC 43300 with and without exposure to the test compounds. For the control, cells exposed to 10% *v*/*v* of DMSO displayed a spherical shape with a smooth cell surface, and several cells underwent cell division (Figure 5a).

The SEM images obviously revealed the concentration-dependent effects of GA and MG on cell morphology. While most bacterial cells treated with either GA or MG at 0.125 × MIC demonstrated divided cells similar to the control, small amounts of the spitting cellular matrix could be observed on the cell surface (Figure 5c,f). Moreover, when the concentrations of GA and MG were increased to 0.5 × MIC, the cellular surface of spherical bacteria remained mostly intact but showed wrinkles and more spitting materials (Figure 5d and Figure 5g, respectively). At the highest concentration of 1 × MIC, both GA and MG caused extensive damage to the cell surface and distorted cell morphology, as illustrated in Figure 5e and Figure 5h, respectively. Notably, regular wrinkles appeared on the cells surface with GA exposure (Figure 5e), whereas MRSA cells treated with MG showed severe cell wall destruction and the leakage of intracellular components, potentially leading to cell lysis (Figure 5h).

To observe the effects of selected combinations on ultrastructural changes, cells were treated at concentrations lower than their MICs. In Figure 5i, when GA and MG were combined at 0.5 × MIC, the bacterial cell walls were severely destroyed, resembling the results seen after exposure to MG at 1 × MIC. This finding suggests the additive effect of GA and MG. As shown in Figure 5b, exposure to ampicillin alone at 0.0625 × MIC caused impairment in most cells, but the addition of either GA or MG at 0.125 × MIC led to further damage to bacterial cells. In Figure 5j,k (red arrows), irregular cell surfaces with dimples and concavities, as well as cell rupture, could be observed after treatment with phenolic combination.

## 3. Discussion

In this study, the antibacterial activity of GA and MG was first evaluated in both MSSA ATCC 25923 and MRSA ATCC 43300. The obtained MIC and MBC values of GA and MG were consistent with our previous study, demonstrating their ability to inhibit both MSSA and clinical MRSA isolates within a concentration range of 0.19 to >3 mg/mL [25]. Furthermore, other studies reported that GA and its alkyl gallate derivatives exhibited similar MIC ranges against various strains of MSSA and MRSA [26,27]. Gobin et al. (2022) found that GA could inhibit the *S. aureus* strain from the Collection de l’Institut Pasteur Paris with MIC of 2.5 mg/mL [26]. The MIC of MG against MRSA ATCC 33591 was similar to our results against MRSA ATCC 43300 strain [27]. In the case of the tested antibiotics, ampicillin, amoxicillin, and penicillin G clearly demonstrated significantly reduced antibacterial efficacy against *S. aureus* ATCC 43300, indicating antibiotic resistance in this strain. *S. aureus* ATCC 43300 contains the β-lactamase gene expression, which is resistant to the β-lactam antibiotics by producing β-lactamase enzymes to hydrolyze the β-lactam ring [28]. Consequently, cloxacillin, a penicillinase-stable antibiotic, showed the highest antibacterial activity among the tested β-lactam antibiotics, whereas other penicillinase-unstable antibiotics demonstrated lower activities against MRSA ATCC 43300. However, the diminished anti-MRSA activities of these antibiotics may also result from other resistance mechanisms in MRSA, such as alterations in penicillin-binding proteins (PBPs) or transpeptidases, and overexpression of efflux pump [29,30].

Based on these findings, although both GA and MG could inhibit MSSA and MRSA, their MIC values were relatively high, approximately > 6-fold higher than those of the β-lactam antibiotics (0.0625–62.5 μg/mL). Consequently, GA and MG may not be effective as monotherapy for MRSA infection. Further studies were conducted to assess the anti-MRSA synergistic effect between the phenolic compounds and the β-lactam antibiotics using three different assays; each method provides its own limitations and advantages for evaluation. The first method involved disc diffusion, which is an affordable and simple technique. In this assay, phenolic compounds and the β-lactam antibiotics were used at fixed concentrations of 10 mg/disc and 10 μg/disc, respectively. The results revealed that GA and MG could not enhance the anti-MSSA activities of β-lactam antibiotics. However, they provided a positive trend in increasing inhibition zone of the β-lactamase-unstable antibiotics against MRSA when compared with antibiotics alone. Additionally, the combination of either GA or MG with only β-lactamase-unstable antibiotics demonstrated identical effects to the phenolic acid alone against MRSA. This suggests that GA or MG may play a pivotal role in the combination to enhance the efficacy of only β-lactamase-unstable antibiotics against MRSA or be the most single active constituent in the combination without antagonizing the antibiotics. It is important to note that the disc diffusion results were determined at a single fixed concentration for each compound. Therefore, the obtained results may not be applicable to the different concentrations. Furthermore, this test only provided qualitative information, which is useful for screening but limited for evaluating synergism, as it may yield controversial results [31,32]. To determine whether synergism or antagonism occurs, it is necessary to conduct checkerboard assay at various concentrations.

The FIC indexes obtained from checkerboard assay against MRSA revealed that MG showed synergism with the β-lactamase-unstable antibiotics, while GA exhibited only an additive effect with the similar drugs, except for cloxacillin. The FIC indexes of cloxacillin, when combined with either GA or MG, indicated an indifferent effect against MRSA. Furthermore, neither of the phenolic compounds could enhance anti-MSSA activities of the tested antibiotics. This finding agrees with a previous report that alkyl gallate could intensify the activity of antibiotic against MRSA. Shibata et al. (2005) reported that FIC indexes of combinations of alkyl gallates (C2–C15) and oxacillin showed synergistic effects against MRSA [33]. In addition, the length of the alkyl chain was also associated with the enhancing activity of oxacillin [33]. In addition to restoring the antibacterial activity of the β-lactam antibiotics, Tamang et al. (2022) found that octyl gallate exhibited synergism with cephalothin, a cephalosporin antibiotic, against some clinical strains of MRSA with FIC indexes of 0.039 [34]. However, octyl gallate showed a lesser degree of decrease in the MIC of other antibiotic classes, such as gentamicin, chloramphenicol, tetracycline, erythromycin, and lincomycin, against MRSA ATCC 33593 [34].

Although a checkerboard assay provides a measure of synergism by assessing the inhibitory activities of antibiotics, this method lacks dynamic information of bacterial death over the time [35,36]. Therefore, a time–killing assay was conducted to observe the synergism of the phenolic compounds and the β-lactam antibiotics. However, because of the labor- and time-intensive nature of this approach [36], the combination of MG and ampicillin, which exhibited the lowest FIC index, was selected for further investigation using this method. The combination of MG and ampicillin at their MIC values was found to suppress MRSA growth to a greater extent than the antibiotic alone after 16 h, resulting in a reduction in bacterial numbers of greater than 2 log_10_-fold compared to ampicillin alone at 24 h. This indicates the synergistic effect. In contrast, this combination was unable to suppress MSSA growth more effectively than a single compound over the course of 24 h. These time–kill results align with the findings of checkerboard study, where the combination of MG and ampicillin exhibited indifference and synergistic effects against MSSA and MRSA, respectively.

Basically, a competitive inhibitor competes with a substrate for binding to the active site of an enzyme, leading to a decrease in substrate activity. Thieme et al. (2018) demonstrated that ampicillin and ceftaroline at high concentrations could compete for binding to PBPs or transpeptidase, resulting in an antagonistic effect against *Enterococcus faecalis* [37]. However, as no antagonistic effect was observed in any of our combinations, the results suggest that the antibacterial mechanism of either GA or MG at tested concentrations may not be associated with the same active site of PBPs, which is the target mechanism of β-lactam antibiotics. In conjunction with the results from the disc diffusion, checkerboard, and time–kill assays, MG showed synergism with ampicillin, amoxicillin, and penicillin G against MRSA, while exhibiting indifferent effects with cloxacillin. It is known that penicillin, ampicillin, and amoxicillin are inactivated by staphylococcal β-lactamases, whereas cloxacillin is relatively stable to staphylococcal β-lactamases [38]. In addition, the production of β-lactamase is one of the resistant mechanisms of MRSA [29]. Therefore, we hypothesized that the synergistic mechanism between MG and the β-lactamase-unstable antibiotics against MRSA may involve bacterial resistance strategies, particularly an association with β-lactamase. Additionally, phenolic compounds could bind with various enzymes, such as amylases, glucosidases, and proteases, resulting in a non-functional complex [39,40]. The molecular docking study conducted by Nithitanakool et al. (2009) demonstrated that the in vitro inhibitory effects on tyrosinase by GA and MG in mango seed kernels could be attributed to the binding of GA and MG in hydrophobic pocket of the enzyme [41]. Therefore, further investigations into the potential mechanism of GA and MG to inhibit β-lactamase were conducted using molecular docking and in vitro tests.

Molecular modelling showed the potential of GA and MG to inhibit β-lactamase; however, their binding scores in the enzyme’s active site were approximately three-fold lower than that of the gold standard compound, clavulanic acid. The docking results were well consistent with the in vitro enzyme inhibition study. In the in vitro test, clavulanic acid could inhibit β-lactamase with greater than 80% relative inhibition. In contrast, MG could only inhibit the enzyme at high concentration of 20,000 μg/mL, while GA could inhibit the enzyme at all tested concentrations. However, the maximum relative inhibition achieved by GA was approximately 45.54% at 20,000 μg/mL.

It is worth noting that the percentage of relative inhibition of MG at 20,000 μg/mL could not be calculated due to high absorbance values. This phenomenon could be explained by interference in the formation of the product. Typically, phenolic compounds and proteins show an intense absorption band at 280 nm, but the formation of phenolic compound-protein complex and pH changes can shift UV absorption peaks toward longer wavelengths (a red shift) [42,43]. This shift may interfere with the measurement of nitrocefin product, which is measured at 490 nm. Therefore, GA and MG were considered to be poor inhibitors of β-lactamase. Notably, the concentration of GA and MG required to inhibit the β-lactamase (20,000 μg/mL) was much greater than their MIC values, approximately 50- and 12.5-fold higher, respectively. This finding suggests the synergistic mechanism of MG in enhancing the anti-MRSA activity of the β-lactamase-unstable antibiotics was poorly associated with β-lactamase.

However, the SEM images of GA and MG revealed concentration-dependent damage to the bacterial cell surface, leading to the leakage of cellular matrix. These results suggest that the anti-MRSA mechanism of both GA and MG may involve the bacterial cell wall. In accordance with our previous study, the mango seed kernel extract, containing pentagalloylglucopyranose, GA, and MG as major constituents, caused leakage from MRSA cells, resulting in a fibrous matrix extending from the treated cell surface [25]. Similar alteration in bacterial cell morphology were observed in Gram-negative bacteria after treatment with GA and MG. Tien et al. (2022) showed that the rising concentration of GA caused an increase number of destructed *Escherichia coli* cells; consequently, bacterial cells exhibited rough surface with the spitting intracellular contents [44]. Additionally, *Salmonella enterica* serovar Typhimurium exposed to MG showed marked membrane disintegration with the leakage of cellular contents [45]. Furthermore, SEM images after treatment with the combination of ampicillin with phenolic compounds also showed the ultrastructural change on the cell membrane with greater severity than the treatment with ampicillin alone, suggesting an enhanced antibacterial effect. The intensified effect of phenolic compounds on the anti-MRSA activity of the β-lactam antibiotic may be similar to the synergistic effect observed between GA and other antibiotic classes against Gram-negative bacteria. Downregulation of mRNA expressions, including acrA, acrB, tolC, acrD and acrF, which are involved in membrane permeability, has been reported as a mechanism of GA in *E. coli*. Consequently, GA could facilitate antibiotic accumulation in bacteria to demonstrate synergism [44]. In summary, the results presented in this study demonstrate that phenolic compounds alone or in combination with the β-lactamase-susceptible antibiotic damage the bacterial cell membrane. In addition, MG was found to increase the anti-MRSA potential of β-lactamase-susceptible antibiotics with poor β-lactamase association. Therefore, it is necessary to examine the combination of MG and the β-lactamase-susceptible antibiotic on clinical MRSA strains and investigate the action of MG on the different bacterial-resistant mechanisms. Additionally, a quantitative experiment to demonstrate the effect of GA and MG on the cell wall, along with investigating the downstream mechanism of action, focusing particularly on the bacterial cell wall and cell membrane component are of interest for future study.

## 4. Materials and Methods

### 4.1. Materials

GA (98.8%) was purchased from EMD Millipore (Buchs, Switzerland). MG (>98%), ampicillin (>98%), amoxicillin (>98%), penicillin G (>98%), and cloxacillin (>98%) were obtained from Tokyo Chemical Industry (Japan). Phosphate-buffered saline (PBS, pH 7.4) was purchased from EMD Millipore. Dimethyl sulfoxide (DMSO) was sourced from Sisco Research laboratory (India). Other chemicals and solvents were of analytical grade and obtained from local distributors.

### 4.2. Bacterial Strains and Culture Conditions

*S. aureus* ATCC 25923 and ATCC 43300 were purchased from the American Type Culture Collection. Bacterial strains were maintained in a mixture of tryptic soy broth (TSB; Difco, Sparks, MD, USA) and 20% *w*/*v* glycerol at −80 °C until use. For experiments, isolated bacterial colonies from the tryptic soy agar (TSA) were cultured separately in TSB at 37 °C overnight (18–24 h). The turbidity of bacterial suspension was adjusted spectrophotometrically at 600 nm to achieve an optical density (OD) of 0.1 and then diluted in TSB at 1:4 ratio (*v*/*v*) to obtain approximately 10^6^ CFU/mL before use.

### 4.3. Antibacterial Susceptibility Test

The antibacterial susceptibility was tested by broth microdilution method to determine the minimum inhibitory concentration (MIC) of each compound. The test compounds were prepared by dissolving in 10% *v*/*v* DMSO in TSB medium. Then, the serial two-fold dilutions of the test compounds were mixed with TSB broth at a 1:1 ratio (*v*/*v*) in 96-well sterile microtiter plates to obtain final concentrations of 12.5–400 μg/mL for GA, 50–3200 μg/mL for MG, and 0.004–250 μg/mL for antibiotics. Overall, 20 μL of the prepared inoculum was added in each well to obtain a 100 μL final volume. The microtiter plates were then incubated at 37 °C for 24 h under aerobic conditions. Negative and positive controls were set in each test. A negative control included the test sample without the bacterial inoculum, while a positive control included the bacteria inoculum without the test sample. The effect of solvent control included the bacteria and replaced the test compounds with 10% *v*/*v* DMSO in TSB medium. The MIC was defined as the lowest concentration at which no bacterial growth is determined by the unaided eye. The growth endpoint in the wells containing test samples was determined by comparing it with the growth in the control wells.

To determine the minimum bactericidal concentration (MBC), a 20 µL of each culture medium from wells with no visible growth was removed and dropped onto TSA plate. After incubation at 37 °C for 24 h, the MBC was defined as the lowest concentration that resulted in no bacterial colony growth. The sample was tested in triplicate in separate experiments.

### 4.4. Enhancing Effects of GA and MG on Anti-MRSA Activity of β-Lactam Antibiotics

#### 4.4.1. Disc Diffusion Assay

The effects of GA and MG on the antimicrobial activity of the β-lactam antibiotics were first assessed via the disc diffusion test. In brief, solutions for all test compounds were prepared in 20% *v*/*v* DMSO in TSB solution. Each prepared bacterial inoculum was swabbed on TSA and allowed to air-dry at room temperature (25 °C). Subsequently, a 6 mm sterile paper disc was loaded with 20 μL of the test solution, resulting in a final content of 10 mg/disc for GA or MG, and 10 μg/disc for the antibiotics. The loaded disc was then placed onto the agar plate, left to dry, and incubated at 37 °C for 24 h under aerobic conditions. A 20% *v*/*v* DMSO solution was used as a negative control. All disc diffusion tests were performed in triplicate, and the antibacterial activity was expressed as the mean of the inhibition diameter (mm).

#### 4.4.2. Checkerboard Assay

The antibacterial synergism among the test compounds was further studied by checkerboard microdilution assay as previously described by our research [46]. A 20 µL of the prepared bacterial inoculum was added into the mixed concentrations of two test compounds, ranging from 0.0625 × MIC to 4 × MIC. These experiments were conducted in triplicate. The FIC index was defined as the lowest concentration of the test combination that resulted in no visible growth of the bacteria. FIC indexes for the dual combinations were calculated using Equation (1).

(1) $$\mathrm{FIC}\;\mathrm{index}=\frac{\mathrm{MIC}\;\mathrm{of}\;A\;\mathrm{in}\;\mathrm{combination}}{\mathrm{MIC}\;\mathrm{of}\;A\;\mathrm{alone}}+\frac{\mathrm{MIC}\;\mathrm{of}\;B\;\mathrm{in}\;\mathrm{combination}}{\mathrm{MIC}\;\mathrm{of}\;B\;\mathrm{alone}},$$

The interpretation of FIC index values was as follows: FIC index ≤ 0.5; synergism, 0.5 < FIC index ≤ 1; additive effects, 1 < FIC index < 2; indifference, and FIC index ≥ 2; antagonism [47].

#### 4.4.3. Time–Kill Assay

The time–kill curves of the selected synergism antibiotic combination were determined according to the method outlined by Gaudereto et al. (2020) with modification [32]. Bacterial suspension was introduced into a TSB broth containing a single compound or a combination at the concentration of MIC. After incubation at 37 °C, the sample was collected at various time intervals (0, 2, 4, 8, 12, and 24 h) and a ten-fold serial dilution was prepared in sterile saline. Thereafter, 20 μL of each dilution was placed on agar plate and incubated at 37 °C for 24 h. A bacterial viability count was performed and recorded as the number of CFU/mL. In each assay, a bacterial growth control comprised the organism and substituted the test compound with a 10% *v*/*v* DMSO in TSB medium. All experiments were carried out in triplicate. Time–kill curves will be established by plotting log_10_ CFU/mL against time. The synergistic, antagonistic, and no difference effects were defined as a  ≥ 2 log_10_ CFU/mL decrease, ≥2 log_10_ CFU/mL increase, and < 2 log_10_ CFU/mL decrease or increase, when compared to the most active agent, respectively [48].

### 4.5. β-Lactamase Inhibition Assays

#### 4.5.1. Molecular Docking Study

The ligands were constructed using Chem3D Pro 12.0: the chemical structures were drawn and energy minimized using the MM2 [49] force field and saved in 3D format. The crystal structure of *S. aureus* β-lactamase was obtained from the Protein Data Bank (PDB) [50] ID:1BLC [51] with resolution 2.20 Å. Discovery studio version 4.5 was used to prepare the crystal structure for docking, i.e., hydrogen atoms were added, and crystallographic solvents and co-crystallized ligand removed. The center of the β-lactamase-binding site was defined at coordinates (x = 5.637, y = −9.664, z = −12.243) with 10 Å radius. The basic amino acids lysine and arginine were defined as protonated. Furthermore, aspartic and glutamic acids were assumed to be deprotonated. The ChemPLP [52] scoring function was implemented for docking the ligands using the Genetic Optimization for Ligand Docking software package (GOLD) version 2022.3.0. Molecular docking was conducted at 100% efficiency at 50 docking runs per ligand.

#### 4.5.2. In Vitro β-Lactamase Inhibition Assay

The β-lactamase inhibitor assay kit (abcam^®^, Cambridge, UK) was used to determine inhibitory activity of GA and MG. Assay was performed in accordance with the manufacturer’s instructions. Briefly, 20 µL of test samples (S) were placed into 96-well microplate with 50 µL of enzyme master mix to give the final concentration of 200, 2000 and 20,000 μg/mL. After pre-incubation at 25 °C for 10 min protected from light, nitrocefin (30 µL) was added and immediately measure absorbance on a microplate reader (Varioskan LUX, Thermo Scientific, Waltham, MA, USA) in a kinetic mode at 490 nm for 15 min. The enzymatic control (EC) was performed without inhibitor and the test compounds.

The clavulanic acid, a positive β-lactamase inhibitor control, was diluted to be 0.002, 0.02 and 0.2-folds from the unidentified original concentration obtained from the company. The experiments were carried out in duplicate, and the rate of the nitrocefin product formation was plotted between average absorbance values and time. The % relative enzyme inhibition was calculated as Equation (2):

(2) $$\%\;\mathrm{Relative}\;\mathrm{inhibition}=\frac{\mathrm{Slope}\;\mathrm{of}\;\mathrm{EC}-\mathrm{Slope}\;\mathrm{of}\;S}{\mathrm{Slope}\;\mathrm{of}\;\mathrm{EC}}\times 100,$$

where slopes of EC and S were calculated in a linear period of 2–10 min.

### 4.6. SEM

The prepared inoculum of *S. aureus* ATCC 43300 was incubated with the test compounds at various concentrations as follows: GA and MG at 0.125, 0.5, and 1 × MICs, ampicillin at 0.0625 × MIC, the combination of GA and MG at 0.5 × MIC, and the combination of ampicillin and phenolic compounds at 0.0625 and 0.125 × MICs, respectively. The bacterial control was performed by replacing the test compounds with 10% *v*/*v* DMSO. After incubation at 37 °C for 12 h, bacterial cells were collected by centrifugation at 4500 rpm for 5 min. Subsequently, 2.5% *w*/*v* of glutaraldehyde in 0.1 M PBS was added to fix the sample overnight. The sample was then applied to a cover slip pre-treated with poly-L-lysine. After 15 min, the cells were washed with PBS and dehydrated using a series of ethanol concentrations (30, 50, 70, 95, and 100% *v*/*v*). After critical point drying and coating with a gold sputter, samples were examined using the SEM (JSM-IT500HR InTouchScope™, JEOL, Tokyo, Japan), with was operated at 15 kV.

### 4.7. Statistical Analysis

All experimental results were expressed as mean ± S.D. All statistical analyses were carried out using SPSS (version 29.0). Analysis of variance was performed by ANOVA. Significant differences between the means were determined using Tukey’s HSD comparison test at a significance level of *p* < 0.05.

## 5. Conclusions

GA and MG exhibited anti-staphylococcal effects against MSSA and MRSA. The extensive damage to cell membranes resulting in bacterial morphological changes consisted of concentration-dependent effects and could be the possible mechanism of action of these compounds. Although GA and MG showed indifferent effects when combined with all β-lactam antibiotics against MSSA, MG displayed marked synergism only with penicillinase-unstable β-lactam antibiotics against MRSA. However, both GA and MG displayed weak inhibitory to the β-lactamases. The synergistic effect between MG and ampicillin was the time-dependent manner and caused damage to the bacterial membrane. Therefore, our study provides beneficial information regarding the potential use of GA and MG as adjunctive antibiotic therapy alongside β-lactams for treating MRSA infection. However, our research included a single standard MRSA ATCC 43300, which may not be applicable to clinical MRSA strains with diverse resistant mechanisms. Further studies of clinical MRSA strains would provide valuable insights into the mechanism of GA or MG-mediated resensitization.

## Figures and Tables

**Table 1:** Antibacterial activities of phenolic compounds and the β-lactam antibiotics against *S. aureus* ATCC 25923 and 43300.

Test Compounds	MSSA *S. aureus* ATCC 25923		MRSA *S. aureus* ATCC 43300	
	MIC (μg/mL)	MBC (μg/mL)	MIC (μg/mL)	MBC (μg/mL)
GA	200	400	400	>400
MG	1600	3200	1600	3200
Ampicillin	0.125	0.25	62.5	250.0
Amoxicillin	0.25	0.5	62.5	250.0
Cloxacillin	0.25	0.5	0.5	1.0
Penicillin G	0.0625	0.125	62.5	125.0
Solvent ^a^	-	-	-	-

^a^: No antibacterial effect could be observed in the test concentrations (0.325 to 10% *v*/*v* DMSO).

**Table 2:** The FIC indexes of the combination of GA, MG, and the β-lactam antibiotics against *S. aureus* ATCC 25923 and 43300.

Bacterial Strains	Compounds	MIC		Combinded Compounds (μg/mL)	MIC		The Lowest FIC Indexes	
		Alone (μg/mL)	Combined (μg/mL)		Alone (μg/mL)	Combinded (μg/mL)		
MSSA (*S. aureus* ATCC 25923)	GA	200	6.25	Ampicillin	0.125	0.125	1.03	IND *
			6.25	Amoxicillin	0.25	0.25	1.03	IND *
			6.25	Cloxacillin	0.25	0.25	1.03	IND *
			6.25	Penicillin G	0.063	0.063	1.03	IND *
	MG	1600	50	Ampicillin	0.125	0.125	1.03	IND *
			50	Amoxicillin	0.25	0.25	1.03	IND *
			50	Cloxacillin	0.25	0.25	1.03	IND *
			50	Penicillin G	0.063	0.063	1.03	IND *
	MG	1600	800	GA	200	25	0.63	ADD *
MRSA (*S. aureus* ATCC 43300)	GA	400	100	Ampicillin	62.5	15.625	0.5	ADD *
			100	Amoxicillin	62.5	15.625	0.5	ADD *
			200	Cloxacillin	0.5	0.25	1	IND *
			200	Penicillin G	62.5	7.813	0.63	ADD *
	MG	1600	100	Ampicillin	62.5	15.625	0.313	SYN *
			200	Amoxicillin	62.5	15.625	0.375	SYN *
			100	Cloxacillin	0.5	0.5	1.06	IND *
			200	Penicillin G	62.5	15.625	0.375	SYN *
	MG	1600	800	GA	400	50	0.63	ADD *

* ADD: additive effect, IND: indifferent effect, SYN: synergistic effect.

## Data Availability

The data are contained within the article.
